# Current Trends and Prospects for Application of Green Synthesized Metal Nanoparticles in Cancer and COVID-19 Therapies

**DOI:** 10.3390/v15030741

**Published:** 2023-03-13

**Authors:** Londiwe Simphiwe Mbatha, Jude Akinyelu, Chika Ifeanyi Chukwuma, Mduduzi Paul Mokoena, Tukayi Kudanga

**Affiliations:** 1 Department of Biotechnology and Food Science, Durban University of Technology, P.O. Box 1334, Durban 4000, South Africa; 2 Department of Biochemistry, Federal University Oye-Ekiti, Private Mail Bag 373, Ekiti State 370111, Nigeria; 3 Centre for Quality of Health and Living, Faculty of Health and Environmental Sciences, Central University of Technology, Private Bag X20539, Bloemfontein 9301, South Africa; 4 Department of Pathology, Pre-Clinical Sciences Division, University of Limpopo, Private Bag X1106, Sovenga 0727, South Africa

**Keywords:** cancer, COVID-19, nanotechnology, metal nanoparticles, green synthesis

## Abstract

Cancer and COVID-19 have been deemed as world health concerns due to the millions of lives that they have claimed over the years. Extensive efforts have been made to develop sophisticated, site-specific, and safe strategies that can effectively diagnose, prevent, manage, and treat these diseases. These strategies involve the implementation of metal nanoparticles and metal oxides such as gold, silver, iron oxide, titanium oxide, zinc oxide, and copper oxide, formulated through nanotechnology as alternative anticancer or antiviral therapeutics or drug delivery systems. This review provides a perspective on metal nanoparticles and their potential application in cancer and COVID-19 treatments. The data of published studies were critically analysed to expose the potential therapeutic relevance of green synthesized metal nanoparticles in cancer and COVID-19. Although various research reports highlight the great potential of metal and metal oxide nanoparticles as alternative nanotherapeutics, issues of nanotoxicity, complex methods of preparation, biodegradability, and clearance are lingering challenges for the successful clinical application of the NPs. Thus, future innovations include fabricating metal nanoparticles with eco-friendly materials, tailor making them with optimal therapeutics for specific disease targeting, and *in vitro* and *in vivo* evaluation of safety, therapeutic efficiency, pharmacokinetics, and biodistribution.

## 1. Introduction

Nanometal therapeutics have transformed the field of nanomedicine over the years. The role that nanodevices have played in the treatment and management of severe diseases such as cancer and coronaviruses is commendable. Cancer is still regarded as one of the most lethal inherited diseases, causing more mortalities than Acquired Immune Deficiency Syndrome, Tuberculosis, and Malaria do globally [1,2]. In 2012 alone, the disease was reported to have killed over 4 million people, and this is presumed to reach over 20 million by 2030 [3]. There are over 100 types of cancer including cancer of the breast, brain, colon, lung, prostate, and skin [4], and the type usually depends on the tissue of origin. Conventional cancer treatments such as chemotherapy, radiotherapy, and surgery are inadequate in treating the disease as they improve the patient’s life, while causing severe side effects [5,6,7], therefore, new and more effective therapies are still urgently needed.

Coronavirus infection disease 2019 (COVID-19) is caused by severe acute respiratory syndrome coronavirus 2 (SARS-Co-2). It is one of the fastest spreading (reaching more than 200 countries) and most lethal infectious diseases worldwide [8]. Its derivative, Middle East Respiratory Syndrome (MERS), was first reported between late 2002 and 2012, and since December 2019, SARS Co-2 has spread exponentially, killing thousands of people globally [9]. According to a recent report, the SARS-CoV-2 virus originated from a bat in Wuhan, China, on December 2019, hence the name COVID-19 [10]. In late January 2020, the World Health Organization declared the virus outbreak a state of health emergency concern. Approximately 40 different strains of SARS-CoV-2 have been reported in recent years [11,12]. Presently, there is still no adequate and effective treatment for the disease. The standard antiviral drugs are associated with non-specific toxicity and drug resistance [13]. The RNA-based vaccines have emerged as promising alternatives due to their simple engineering and ability to target structural viral proteins triggering the production of antibodies that neutralize the virus [14]. They are, however, associated with some unwanted immune responses [15], thus, more sophisticated, site-specific, and safe antiviral agents, drugs, and vaccines are still needed to fight this disease. To overcome the above-mentioned limitations and advance both anticancer and antiviral treatments, a combined effort of multidisciplinary research fields is needed to develop alternative anticancer and antiviral drugs/vaccines.

Metal nanoparticles (NPs), particularly those formulated through green nanotechnology, have gained much attention as cancer or viral therapeutics and therapeutic drug delivery systems because of their biocompatibility, scalability, cost effectiveness, eco-friendliness, outstanding physiochemical properties, antiviral properties, and anticancer properties [16,17,18]. In cancer or COVID-19 treatments, it has been reported that combining metal NPs with a therapeutic drug has demonstrated significant potential in treating these diseases [19]. The metal NPs, if they are green synthesized, elicit their intrinsic anticancer or antiviral effects and improve the therapeutic drug efficiency by improving its pharmacokinetics through enhancing its circulation half-live and reduction of its side effect [20]. Additionally, it is believed that the nano size of metal NPs allows them to easily cross cellular membranes and accumulate in the tumour sites through passive targeting [21]. In the case of viral disease treatment, the nano size of metal NPs enables them to target various sites of the virus life cycle and inactivate the virus, inhibit viral production or replication [22,23]. Several metal NPs are used as diagnostics, therapeutics, or drug delivery systems [24,25,26]. Other examples of metal NP-coated drugs in clinical trials or in the market include AurImmune, an AuNP conjugated with thiolated polyethylene glycol and tethering tumour necrosis factors alpha (TNFs-α), which is under investigation in phase III clinical trials for treatment of advanced cancers [27,28], and Auronafin, a AuNP conjugated with triethyl phosphine, which is an FDA-approved drug (for the treatment of rheumatoid arthritis) that has been reported to be effective against SARS-CoV-2 infection [29].

Therefore, this review highlights the recent developments of metal NPs formulated using nanotechnology strategies for cancer and COVID-19 preventions and treatments, as well as the issues pertaining to their applicability in clinical studies.

## 2. Nanotechnology

The field of nanotechnology has played a massive role in the advancement of many anticancer nanotherapeutics over the years and has received much attention already in the investigations of novel antiviral nanotherapeutics [30,31,32]. Nanotechnology is amongst the fastest growing multidisciplinary fields due to its vast array of applications in medicine. It falls under the field of nanomedicine, which involves the use of nanomaterials/nanostructures for the control, diagnosis, prevention, and treatment of many diseases [33]. It comprises nanostructures and nanomaterials that encompass the fabrication of chemical, physical, and biological nanosystems (<100 nm) and their application in larger-scale systems [34]. The technology provides an opportunity to fine-tune the properties of nanostructures and nanomaterials at the atomic, molecular, and macromolecular levels to produce nanosystems with enhanced physicochemical properties, thus, attracting large-scale research interest. These nanosystems include nanotubes, nanowires, nanorods, and NPs.

Over the years, NPs, particularly, metal NPs, have been extensively exploited for the early diagnosis and treatment of diseases due to their remarkable properties including tuneable shape, small size, high drug loading capacity (due to high surface–volume ratio), paramagnetic cloud, improved solubility, improved stability, ability to cross blood-brain or air barriers, bioavailability, photon conversion, surface adaptability, biocompatibility, and biodegradability [35], as well as innovative approaches in their synthesis. It is believed that these unique features make the NPs more effective due to the possibility of improving their interaction with the surrounding environment [36]. In cancer treatment studies, NPs, such as gold (Au) [37], silver (Ag) [38], platinum (Pt) [39], CeO_2_ [40], zinc oxide (ZnO) [40], titanium dioxide (TiO_2_) ones [41], have been used and found to be effective. Their anticancer activity is said to be due to the pro-oxidant behaviour observed inside the cancer cells. Once inside the cells, these NPs interact with the cellular matrix, which leads to the disturbance of various signalling pathways. This causes an increased influx of free radicals, leading to oxidative stress and cell death [42]. The therapeutic potential of NPs has also been documented for chronic and non-chronic infectious diseases such as Human immunodeficiency virus (HIV), Herpes simplex, Influenzas, Respiratory syncytial virus (RSV), Zikavirus, and Smallpox [43]. NPs of TiO_2_, Ag, ZnO, and Au have been used and found to be effective. Some of the effects of these metal NPs and their salts or nanocomplexes on influenza-like COVID-19 include the prevention of the binding and entry of the virus into the cells [8], the inhibition of viral RNA production and viral replication [44], the enhancement of the inflammatory responses [43], T cell stimulation and activation, and the inhibition of helix uncoupling and SARS-CoV-2 nsp13 ATPase activation [45]. The former one demonstrates the potential nanomedical applications of these NPs in the treatment of cancer and COVID-19. However, before the nanomedical applications are outlined, it is pertinent to mention the various innovative approaches used to synthesize them.

## 3. Synthesis of Metal Nanoparticles

Metal NP synthesis usually involves several approaches. They can be grouped into two main types, namely top-down synthesis and bottom-up synthesis, which primarily differ from each other in terms of the starting materials used [46]. The top-down synthesis involves the size reduction of bulk starting materials using various chemical and physical treatments such as laser ablation, thermal processing, and mechanical milling [46,47]. Though this method is easy to perform, its main drawback is that it affects the surface chemistry and physicochemical properties of the emanating NPs [46,47]. Bottom-up synthesis or building-up synthesis, on the other hand, involves the preparation of NPs by combining smaller particles such as atoms/molecules [46]. Here, the building blocks of NPs are initially produced, and then combined to form the final NPs [46,48]. Some of the examples of this method include physical or chemical vapor deposition, sol-gel, chemical reduction, solvothermal synthesis, and pyrolysis (spray, laser, and flame), and amongst these, chemical reduction is commonly used [49]. Though this approach produces NPs with unique and attractive physicochemical properties, its continued application, especially in nanomedicine, is limited by the expensive equipment used, excessive labour, controlled reaction conditions, hazardous chemicals used, toxic by-products, and lack of large-scale productivity [50,51]. Thus, there has been an urgent need for an alternative simple, eco-friendly, environmentally friendly, non-toxic approach in this context. To this end, the green synthesis of metal NPs has emerged.

### Green Synthesis as An Ideal Approach

Green synthesis utilizes the bottom-up approach and involves the production of NPs using microorganisms, such as algae, fungi, bacteria, plants, human cell lines, and biocompatible biomolecules [52,53,54]. The production of NPs using microorganisms is achieved by intracellular or extracellular synthesis based on the location where the NPs are produced. The process is facilitated by extracellular or intracellular biomolecules or enzymes. Intracellular microbial synthesis involves the transportation of metal ions into the microbial cells to form NPs through intracellular enzymes. The extracellular microbial synthesis involves the accumulation of metal ions outside the microbial plasma membrane, followed by the reduction of the ions through extracellular enzymes [55]. In microbial systems, metal ions are detoxified through direct redox reactions [56], volatilization via ethylation or methylation [17], for the creation of phosphate, sulphide, carbonate, and phytochelatin (metal ions binding to peptides) derivatives. However, there are challenges associated with the synthesis of NPs using microorganisms, which limit or discourage their application. They include the difficulty of obtaining the final product, the cost of isolated microorganisms, the maintenance of optimized microbial growth conditions, and the formulation of corresponding biomass, complex and expensive cell culture media used, and issues of possible aggregation of the NPs produced [57].

**Figure 1:**
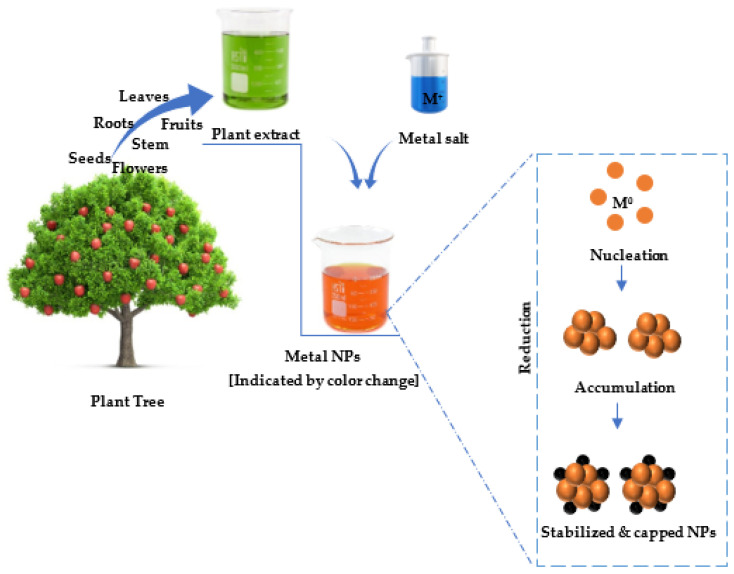
Synthesis of metal NPs using plant extracts.

The production of metal NPs using plants is achieved through the reduction of metal ions using plant extracts under set conditions. The synthesis procedure is conducted in three steps: (1) the activation phase, where the metal ions undergo reduction by the plant extracts’ phytoconstituents before undergoing nucleation; (2) the growth phase, where NPs assemble to form larger-sized NPs; (3) the termination phase, where the final NPs with a specific shape are produced [34] (Figure 1). This approach is simple, cost effective, uses benign solvents and non-hazardous capping/stabilizing agents and is environmentally friendly [17]. The metal NPs produced by this method usually possess high surface functionalities, which presents a high possibility of agglomeration. Hence, capping agents (e.g., natural polymers) are usually used to avoid agglomeration and to control the final size of the NPs.

For decades, plants have been used to treat many diseases from mild flus and inflammations to severe diseases such as cancer and viral infections. The medicinal properties of plants have been reported to be due to the presence of various bioactive compounds such as phenolic acids, flavonoids, polyphenols, alkaloids, and terpenes [20,58,59]. For instance, flavonoids such as quercetin, rutin, kaempferol, and myricetin, as well as alkaloids such as avicins, asmatrine, boswellic acids, fomitellic acids triterpenoids, pomolic acids, oleanolic acids, sanguinarine, and ursolic acids, have been reported to possess anticancer properties [16,58]. Moreover, flavonoids such as naringenin, myricetin, quercetin-3-rhamnoside, puerarin, quercetin, kaempferol-7-O-glucoside, luteolin, apigenin, vitexin-2-O-rhamnoside, vitexin, and epigallocatechn-3-gallate have been shown to possess antiviral properties [18], while alkaloids such as ajacisine C, isodelectine, ajacisine E, and ajacisine D have been reported to possess anti-respiratory syncytial viral activities [60,61]. During the plant-mediated synthesis of metal NPs, the bioactive compounds, particularly, phenols and flavonoids act as both reducing and stabilizing/capping agents [62]. For instance, flavonoids possess many functional groups with the great capacity to reduce metal ions. Herein, the reduction of metal ions into NPs is facilitated by keto-enol tautomerism [63]. The production of NPs with this approach is faster compared to the speed of microbial-mediated synthesis [53]. The NPs are more stable, well dispersed, and non-toxic [64]. To maintain sustainability, the use of biomass plant extracts as starting materials has been recommended [65]. It is noteworthy that plant-mediated synthesis of metal NPs follow the twelve principles of green chemistry [66], and thus, are aligned with a safe and sustainable future. Moreover, the synthesized NPs possess enhanced medicinal properties, which can be further explored for their potential relevance in cancer and COVID-19 treatments.

## 4. Metal Nanoparticles in Cancer Treatment

Cancer is commonly caused by internal factors such as genetic mutations that occur during normal cell growth and division, which lead to unregulated cell division and the overgrowth of abnormal cells, causing masses of tumours [2]. These genetic mutations cause the upregulation of proto-oncogenes, which then signals the downregulation of tumour suppressor genes that control cell division and growth (carcinogenesis). Due to these mutations, the formed abnormal tumour cells possess unique properties such as replication superiority, enhanced expression of growth signals, unregulated cell division, and the ability to invade neighbouring tissues and avoid apoptosis [67]. Conventional cancer treatments, such as chemotherapy, radiotherapy, and surgery, are inadequate in curing this disease as they improve the patient’s life, but cause severe side effects [19,68]. For instance, the intravenous administration of anticancer drugs during chemotherapy usually leads to unwanted systemic toxicities since the drugs targets both tumours and non-tumour/healthy cells. Additionally, the repeated use of anticancer drugs usually results in the development of resistance, also known as multidrug resistance (MDR) [69]. The therapeutic efficiency of these drugs, when they are used alone, is also not impressive due to the systemic degradation that occurs before they reach the desired site, which ultimately lowers their bioavailability. Thus, safe, efficient, and site-specific anticancer drugs/nano cancer therapeutics may be better alternatives. Metal NPs, particularly, metal and metal oxide NPs produced by green synthesis, have emerged as potentially preferred alternative anticancer therapeutics, diagnosis agents, or anticancer drug delivery nanosystems due to their unique photocatalytic, optical, electrical, and magnetic properties, eco-friendliness, non-toxicity, cost-effectiveness, non-hazardousness, natural antioxidant effectivity, superior physicochemical features, biodegradability, biocompatibility and amenable surface that is capable of conjugating with drugs/genes and various targeting moieties [70].

### 4.1. Anticancer Properties of Metal Nanoparticles and the Possible Mechanisms of Action

Metal NPs have been reported to easily enter the mammalian cancer cells via various mechanisms such as phagocytosis, pinocytosis, and endocytosis due to their unique tuneable physicochemical properties. It is said that the small NPs enter the cell through caveolin-receptor-mediated uptake pathways, while the large/bulk/aggregated NPs enter through clathrin receptor-mediated endocytosis or phagocytosis uptake mechanisms. It is believed that upon binding to the cell membrane, the NPs are taken up by one of the aforementioned pathways. Once they are inside the cell, they either interact with intercellular proteins or they enter the endosome vacuole, and subsequently the lysosome vacuole, where they either become degraded, altered, or dissociated before being released into the cytosol [31]. Inside the cytosol, the NPs bind with the reactive oxygen species (ROS) and release the metal ions that bind to the cytosol proteins, altering their morphology. This affects the physiology of the cell, triggering different programmed cell deaths [71]. Apoptosis and necrosis are the two main programmed cell deaths discussed herein. Understanding these mechanisms helps in gaining an insight into the cancer pathophysiology, which may be helpful in the development and advancement of agents/drugs/vaccines that can target specific sites of these mechanisms.

**Figure 2:**
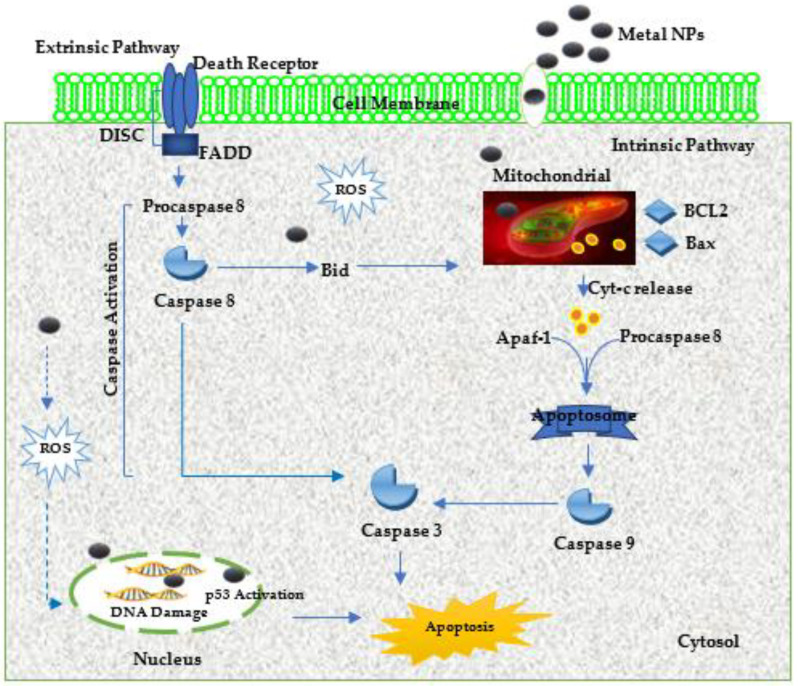
Schematic representation of the metal nanoparticles’ anticancer mechanism of action via extrinsic and intrinsic apoptosis pathways.

Apoptosis is programmed cell death/suicide that is predominantly driven by caspase enzymes, which are both initiators and executioners of this pathway. NPs can trigger this mechanism intrinsically (on the mitochondria) or extrinsically (on the death receptor) (Figure 2). Intrinsic apoptosis is activated by intracellular stress caspase-independent or caspase-dependent mechanisms. Along this pathway, the generated ROS cause conformational alterations of the mitochondrial membrane, which triggers the release of cytochrome-c (Cyt-c) in the cytosol. The released Cyt-c binds with pro-caspase-8 and apoptotic protease factor-1 (Apaf-1), activating the caspase-9/3 apoptotic mechanism, which initiates apoptosis by cleaving the cytoplasmic and nuclear substrates (Poly ADP-ribose Polymerase-1 or PARP-1). The extrinsic apoptosis pathway/death receptor pathway is initiated when death ligands such as TNF and Fas ligand (FasL) attach or bind to the death receptors, including type 1 TNF receptor TNFR1 and Fas (CD95) [72], which comprise of an intracellular death domain that is capable of recruiting adapter proteins such as Fas-associated death domain (FADD), TNF receptor-associated death domain (TRADD), and cysteine proteases [73,74]. The binding results in the development of a ligand-adaptor protein binding site known as the death-inducing signalling complex (DISC) [74,75], which signals the activation of pro-caspase-8, an initiator caspase that starts apoptosis by cleaving other caspases (downstream or executioners) [74].

Necrosis is another programmed cell death mechanism, in which ligands, including FasL, TNF-related apoptosis-inducing ligand (TRAIL), and TNF-α, bind to their respective receptors to form a complex comprising Fas-associated protein with death domain (FADD), caspase-8, and receptor-interacting protein-kinase-3 (RIP3). The interaction of this complex with metabolic enzymes triggers the production of ROS, which causes necrosis. Other cell-death initiators/mediators such as PARP, PARP-1, RIP kinase, and calpains also become triggered during stressful conditions. During necrosis, NPs signal the activation of RIPI3 and RIPI, which affects the mitochondria by increasing the production of NADPH (Nicotinamide adenine dinucleotide phosphate) oxidase and cellular calcium (Ca^2+^), thus triggering ROS production and influx and inducing programmed necrosis [74]. The cellular morphological changes caused by apoptosis/necrosis include the loss of membrane integrity, nuclear fragmentation, chromatin condensation, cytoplasmic shrinkage, and structural configuration of cytoplasmic organelles, which can be observed microscopically [74,76]. The activation of both these mechanisms is dependent on the type of cell, as well as the size and dose of the metal NPs applied [77].

Gold NPs as Anticancer Agents: Gold NPs (AuNPs) have been the most studied metal NPs in nanomedicine since their discovery centuries ago due to their high surface area-to-volume ratio, tuneable physicochemical properties, and optoelectronic features [78]. Their nano-meter sizes specifically allow them to accumulate in tumour sites and transfect cells quickly using different pathways [79]. Over the years, various studies have demonstrated the anticancer effect of AuNPs generated using green chemistry on different cancer cell lines (Table 1). For example, Jeyaraj et al. [80] synthesized AuNPs using a *Podophyllum hexandrum* L. water-based leaf extract. These NPs were generally spherical, had a range of 5–35 nm diameter, and showed a DNA damage-triggered anticancer effect on HeLa, with an IC_50_ of 20 μg/mL. Similarly, Klekotko et al. [81] fabricated AuNPs using *Mentha piperita* water-based leaf extract; the NPs were spherical, hexagonal, triangular, had a diameter range of 10–300 nm, and demonstrated a cytotoxicity effect on HEK293 cells. Tiloke et al. [82] also fabricated AuNPs using a *Moringa oleifera* water-based leaf extract; the NPs were polyhedral and spherical, had a diameter range of 10–20 nm, and showed a caspase-mediated apoptosis triggered anticancer effect on cancerous cell lines A549 and SNO with IC_50_ values of 98.46 and 92.01 μg/mL, respectively, with minimal effect on noncancerous cells. Patil and co-workers [83] produced AuNPs using a *Rhus chinensis* water-based galls extract; the NPs were oval and spherical, ranged from 20–40 nm in diameter size, and demonstrated DNA damage stimulated cytotoxicity effect against Hep3B, MG-63, and MKN-28 cancer cells, with an IC_50_ value of 150 µg/mL. Barai et al. [84] fabricated AuNPs using *Nerium oleander* stem bark extract; these NPs were mostly spherical, while some were hexagonal, triangular, rod-like, and flower-like ones, the NPs had a range of 10–100 nm in size, and showed significant anticancer activity against human breast cancer cells (MCF-7 cell line), with an IC_50_ value of 74 μg/mL. Lui et al. [85] produced AuNPs using a *Curcuma wenyujin* extract; the NPs were spherical, were 200 nm in size, and showed a significant apoptotic effect against human renal cell carcinoma A498 and SW-156 cells, with IC_50_ values of 25 µg/mL and 40 µg/mL, respectively. The apoptotic activity against A498 was found to be via ROS production, mitochondrial dysfunction, and caspase cascade-mediated apoptosis. El-Borady and co-workers [86] biosynthesized AuNPs using *Petroselinum crispum* Presley leaf extract; the NPs were spherical, semi-rod aggregates, and flower-shaped ones, and the NPs ranged from 20–80 nm and showed significant anticancer activity on the human cancerous colorectal cell line (CO-II), with the IC_50_ value of 84.39 µg/mL. Alghuthaymi et al. [87] fabricated AuNPs using *Polianthes tuberosa* L. floral extract; the NPs were exhibited with various shapes including triangles, spheres, rods, hexagons, and pentagons, the NPs ranged from 10–100 nm in particle size, and they elicited significant ROS production and had a caspase cascade-mediated apoptosis triggered cytotoxicity effect on MCF-7. Hosny et al. [88] synthesized AuNPs using the aqueous extract of *Tecoma capensis* leaves; the NPs were spherical and had a range of 10–35 nm in size, demonstrating excellent anticancer activity against MCF7 cell line, with IC_50_ values of 9.6 µg/mL. Various other studies have been reported over the years [89,90,91,92,93,94,95,96,97,98,99,100,101,102,103,104,105,106,107,108].

Silver NPs as Anticancer Agents: Silver NPs (AgNPs) are also among the most studied in biomedical applications, mostly due to their potent antimicrobial activity and unique physicochemical properties, which enhance their efficiency [42]. In recent years, several studies have demonstrated the anticancer activity of green synthesized AgNPs against various cancer cell lines *in vitro* (Table 2). Mukundan et al. [109] biosynthesized AgNPs using *Bauhinia tomentosa* Linn (Kanchini) leaves extract; the NPs were spherical, polydisperse, and were 16.73 nm in diameter size; the NPs showed anticancer activity against lung A-549 carcinoma cell line, with an IC_50_ value of 28.125 μg/mL. In another study, Salehi et al. [110] produced AgNPs using *Artemisia marschalliana* aerial extract; the NPs were predominantly spherical, had a range of 5–50 nm in size, and showed anticancer activity against human gastric carcinoma AGS cell line, with an IC_50_ value of 21.05 µg/mL. Kelkawi et al. [111] synthesized AgNPs using *Mentha pulegium* aqueous and methanolic (MeOH) extracts; the NPs were monodispersed, mostly anisotropic, and had a range of 5–50 nm in size; the NPs showed considerable anticancer activity against HeLa and MCF-7 cancer cells, with IC_50_ values of approximately 100 µg/mL. Palem et al. [112] fabricated AgNPs using *Rheum Rhabarbarum Rhubarb* aqueous stem extract; the NPs were spherical and had a range of 5–50 nm in size; the NPs triggered certain anticancer signalling mechanisms, causing a rapid inhibition of HeLa cell proliferation/viability, with an IC_50_ value of 10 mg/mL. Erdogan et al. [113] formulated AgNPs using the *Cynara scolymus* (Artichoke) aqueous leaf extract; the NPs presented as spherical aggregates with a size range of 200–223 nm; the NPs demonstrated broad-spectrum anticancer activity against MCF7 breast cancer cells, with an IC_50_ value of 10 μg/mL, which was promoted by ROS production, mitochondrial dysfunctioning, and apoptosis cascade proteins activation. Venkatadri and co-workers [114] formulated AgNPs using *Curcuma longa* and *Zingiber officinale* aqueous rhizome extracts: the NPs were spherical, had a size of 42–61 nm, and showed anticancer activity against human colon carcinoma (HT-29), cells with an IC_50_ value of 150.8 µg/mL. Alahmad et al. [115] formulated AgNPs using *Hypericum Perforatum* L. (St John’s wort) aqueous extract; the NPs were monodispersed, spherical, and had a range of 20–50 nm in size; the NPs demonstrated high anticancer activity against HeLa (IC_50_ = 6.72 μg/mL), HepG2 (IC_50_ = 6.88 μg/mL), and A549 cells (IC_50_ = 6.08 μg/mL). In another study, Ramdath and co-workers [42] formulated AgNPs using *Cowpea* starch aqueous seed extract; the NPs were spherical, had a range of 40–70 nm in size, and demonstrated anticancer activity against HEK293, MCF-7, and A549, with IC_50_ values of 41.7, 56.3, and 63.8 μg/mL, respectively. Lastly, Abdellatif et al. [116] synthesized AgNPs using *Allium cepa* L. (*A. cepa*) aqueous extract: the NPs were cubic, had a range of 150–250 nm in size, and showed anticancer activity against human colorectal cancer cell lines (HT-29 and SW620). Several studies of various green synthesized AgNPs and their anticancer activity against various other cell lines of lymphoma [117], lung [118,119,120], ovarian [121,122], melanoma [123], prostate [124], and pancreatic [125] cancers have been reported over the years.

Magnetic NPs as Anticancer Agents: Magnetic NPs (MNPs) show great promise in many cancer applications such as hyperthermia, MRI imaging, anticancer therapeutics, and drug and gene delivery nanosystems [126]. These are nano-sized (<100 nm) metal clusters that operate under an external magnetic field, which can be directed to target a specific tissue and be removed upon completion of therapy. MNPs are superparamagnetic, meaning that they do not retain magnetization once the electric field has been removed, hence presenting an advantage of reducing particle aggregation [127]. The commonly used MNPs include magnetite (Fe_3_O_4_), cobalt ferrite (FeCoO_4_), maghemite (γ-Fe_2_O_3_), chromium dioxide (CrO_2_), and nickel oxide (NiO) [126]. Among these, iron oxide NPs (IONPs) (Fe_3_O_4_) are commonly used in biological applications due to their stability (post coating), lower toxicity, biocompatibility, and biodegradability [128]. Though the use of these NPs, particularly, the green-formulated ones, is still in its infancy, they hold great potential as anticancer therapeutics, as demonstrated by several studies over the years (Table 3). Earlier, Namvar and colleagues [129] formulated magnetic iron oxide nanoparticles (Fe_3_O_4_ MNPs) using *Sargassum muticum* brown seaweed aqueous extract; the NPs were cubic with a mean particle size of 18 ± 4 nm. The MNPs exhibited significant caspase cascade production-mediated anticancer activity against human cell lines for breast cancer (MCF-7 cells), leukaemia (Jurkat cells), liver cancer (HepG2 cells), and cervical cancer (HeLa cells), with IC_50_ values of 18.75 μg/mL, 6.40 μg/mL, 23.83 μg/mL, and 12.50 μg/mL respectively. Later, Nagajyothi et al. [130] synthesized Fe_2_O_3_ NPs using *Psoralea corylifolia* aqueous seeds extract: the NPs were spherical, rod-like, hexagonal, cubic, and crystalline in shape, with a mean diameter size of 39 nm. The NPs showed significant caspase-3 cascade stimulated apoptosis/anticancer activity against renal carcinoma cells (Caki-2 cells), with an IC_50_ value of 0.8 mg/mL. Farshchi and co-workers [131] fabricated FeNPs using *Rosmarinus officinalis* L. aqueous extract; the NPs were mostly spherical, with a mean size of about 100 nm. The NPs showed significant anticancer activity against mice breast cancer cell line 4T1 and mice colon cancer cell line C26, with IC_50_ values of 44 µg/mL and 100 µg/mL respectively. In 2020, Yusefi et al. [132] biosynthesized Fe_2_O_3_ NPs using *Punica granatum* fruit peel extract; the NPs were mostly spherical, while some were cubic, with a hydrodynamic size of 26.52 nm. The NPs showed potent anticancer activity against nasopharyngeal carcinoma (NPC) cell line and HONE1, with IC_50_ values of 197.46 and 85.06 μg/mL respectively. Sarala et al. [133] synthesized magnetic spinel zinc ferrite nanoparticles (ZnFe_2_O_4_ NPs) using *Lawsonia inermis* aqueous leaves extract; the NPs were nearly spherical and rectangular with an average particle size of 45.84 nm. The NPs demonstrated a good ROS generation-mediated anticancer activity against breast cancer (MCF-7) cell lines. In 2021, Yoonus et al. [134] formulated α-Fe_2_O_3_ using *Piper betel* leaves extract; the NPs were cubic and had a mean diameter size of 25.17 nm. The NPs demonstrated significant anticancer activity against lung cancer A549 cells, with an IC_50_ value of 104.6 mg/mL. Yusefi et al. [135] also formulated Fe_3_O_4_ NPs using *Garcinia mangostana* fruit peel crude extract; the NPs were spherical with a mean diameter size of 13.42 ± 1.58 nm and hydrodynamic sizes of less than 177 nm. The NPs displayed anticancer activity against HCT116 colon cancer cells and CCD112 colon normal cells, with IC_50_ values of 99.80 µg/mL and 140.80 µg/mL respectively. Ansari and Asiri [136] fabricated fatty acids-mediated superparamagnetic maghemite nanoparticles (γ-Fe_2_O_3_ NPs) using aqueous polyherbal drug Liv52 (L52E) extract; the L52E-γ-Fe_2_O_3_ NPs were generally spherical, agglomerated, irregular, and spongy with uneven surfaces; the NPs had a mean particle size of 30.66 nm and displayed potent anticancer activity against human colorectal (HCT116) cancer cells. Furthermore, owing to their magnetic property, these NPs can be further enhanced by conjugating them with other therapeutic drugs. Other reported studies of various green synthesized IONPs with anticancer properties include [137,138,139,140,141,142].

Titanium Oxide NPs as Anticancer Agents: Titanium Oxide NPs (TiO_2_ NPs) are semiconductors that possess a large bandgap energy of approximately 3.2, making them capable of generating electron-hole pairs, which interact with the moisture and atmospheric oxygen to produce ROS [143]. These ROS can bind with cell membranes, triggering cell death. These NPs are stable, bio-friendly, cheap to produce, low in toxicity, and possess high photoactivity [144]. Some of the biomedical applications of TiO_2_ NPs include applications such as photodynamic therapeutics [145], diagnostic agents [146], and efficient biocompatible anticancer therapeutics/delivery nanosystems [147]. Several studies have demonstrated the anticancer effect of these eco-friendly NPs as agents (Table 4). Renuka and Soundhari [148] formulated TiO_2_ NPs using *Terminalia chebula* fruit rind extract; the NPs were irregular in shape and had a range of 80–100 nm in particle size. The NPs significant exhibited anticancer activity against A549 cell lines, with an IC_50_ value of 62.5 μg/mL. He et al. [149] produced TiO_2_ NPs using *Cinnamomum tamala* leaf extract; the NPs were irregular, with a mean diameter size of 23 nm. The NPs exhibited dose-dependent anticancer activity against the human prostate cancer (D145) cell line. Hariharan et al. [150] synthesized titanium dioxide (TiO_2_) and palladium (Pd)-doped TiO_2_ (Pd@TiO_2_) NPs using aloe vera gel; the NPs were irregular, while the Pd NPs presented with tiny dots on the surface; the Pd NPs were measured to be 11 nm in particle size. Both the Pd@TiO_2_ NPs and TiO_2_ NPs displayed significant anticancer activity against A549 cell lines, with IC_50_ values of 165 μg/mL and 210 μg/mL, respectively. Rao et al. [151] formulated silver-doped TiO_2_ nanoparticles (Ag/TiO_2_) using *Acacia nilotica* aerial aqueous extract; the NPs were spherical, had a range of 20–40 nm in size, and demonstrated great ROS generated mediated anticancer activity against MCF-7 cells. In 2020, Rehman and co-workers [152] formulated TiO_2_ NPs using Fomes fomentarius intracellular wild mushroom extract; the NPs were irregular aggregates with a particle size range of 100–120 nm. The NPs exhibited anticancer activity against human colorectal carcinoma cells (HCT-116) with significant morphological modifications/damage. Sekimukai et al. [153] fabricated TiO_2_ NPs using the *Withania somnifera* root extract; the NPs were spherical aggregates, with a size range of 50–90 nm. The NPs showed ROS production-mediated anticancer activity against HepG2 cells, with an IC_50_ value of 53.65 µg/mL. Finally, Aswini et al. [154] synthesized TiO_2_ NPs using a *Ledebouria revoluta* bulb aqueous extract: the NPs were spherical, tetragonal, and had a mean crystalline size of 47 nm. The NPs exhibited great anticancer activity against human lung cancer cell lines (A549), with an IC_50_ value of 53.65 µg/mL.

The use of anticancer drugs such as doxorubicin is usually associated with adverse side effects on normal cells, thus, safe, and effective alternative cancer therapeutics are needed, and to address this, many of the research studies have focused on green synthesized metal NPs as potential alternatives. Therefore, from the catalogue of the previously reported studies, it can be highlighted that the anticancer activity of the green-synthesized metal NPs is due to the presence of various bioactive compounds such as flavonoids and alkaloids presented by the plant extract, which possess anticancer properties, as well as the morphology, size, and distribution of metal NPs. The nano-meter sizes, particularly, allow them to easily cross cellular membranes and accumulate in tumour sites through passive targeting and transfect cells quickly using different pathways [21]. Furthermore, in other studies, the anticancer activity of the green-synthesized metal NPs (e.g., AgNPs formulated from coffee extract) was compared to that of doxorubicin conjugated metal NPs on selected non-cancerous and cancerous cells [155]. The results showed that the green synthesized metal NPs displayed more biocompatibility for normal cells and higher cytotoxicity for cancer cells compared to those reported for chemical synthesized metal NPs. Further, the results also demonstrated that the doxorubicin conjugated metal NPs had a reasonable cytotoxic effect against cancer cells with a minimum toxic effect on normal cells. Overall, these studies highlight the potential of green-synthesized metal NPs as safe and effective alternative cancer therapeutics.

### 4.2. Metal Nanoparticles as Drug Delivery Systems

For decades, gene therapy and chemotherapy have been used to combat the fast-growing cancer diseases. However, issues of safety and efficiency still limit their clinical use. Cancer gene therapy involves the integration of new genes with nucleic acids such as plasmid DNA and messenger RNA to replace the dysfunctional ones or by silencing the expression of the diseased genes using the small interference RNA (siRNA) through the RNAi mechanism [67]. Chemotherapy, on the other hand, involves the use of FDA-approved anticancer drugs to treat cancer. But the direct application of a bare gene/drug into the diseased cells is associated with many issues, including poor bioavailability due to possible degradation by intracellular enzymes, limited nuclear uptake due to their enormous size and charge, and toxicity due to the induction of unwanted immunogenicity. To evade this, smart targeted drug/gene delivery systems must be developed. The standard features of an ideal drug/gene delivery system for biological applications include non-toxicity, eco-friendliness, the ease of formulation, biocompatibility, biodegradability, those possessing a large carrying capacity, the ability to protect the therapeutic cargo from degrading enzymes, those possessing a nano-size that can traverse biological membranes, and the ability to be specific to deliver the therapeutic cargo to the target site [67,156].

Metal NPs have proven to be beneficial in cancer treatment as efficient gene/drug delivery systems due to their unique characteristics including low toxicity, nano size, biocompatibility, large surface area, and optical properties [156]. It is said that by manipulating the surface chemistry, size, and the materials used to produce the metal NPs, sophisticated and stable versions of these NPs can be designed to encase therapeutic drugs, genes, and agents and deliver them effectively to specific sites with minimal side effects [157]. It is believed that the nano size of metal NPs allows them to easily cross cellular membranes and accumulate in the tumour sites through passive targeting [21]. Also, the large surface-area-to-volume ratio of the metal NPs facilitates the bioconjugation or loading of different therapeutic cargo such as proteins, drugs, and genes. The surface chemistry of metal NPs allows for the bioconjugation of biomolecules such as polymers, polyethylene glycol derivatives, lipids, peptides, and targeting moieties. Cationic polymers, for example, increase the therapeutic cargo loading capacity, facilitate the binding of charged drugs/genes with the metal NPs, and subsequently, condensation into nanocomplexes that protect the therapeutic cargo against degrading cellular enzymes [19]. Cationic polymers also ensure the binding of nanocomplexes with anionic cell surface, facilitating efficient cellular internalization; they enhance the cytoplasmic circulating time of the nanocomplexes, as well as ensure sustained and controlled drug/gene release [158,159]. Tailoring the metal NPs with targeting ligands such as peptides, lipids, antibodies, aptamers, etc., that are abundantly situated on the surfaces of various cancer cells [156,160] ensures specific delivery of the therapeutic cargo to the desired sites, thus eliminating the unwanted side effects.

Several researchers have demonstrated the ability of various metal NPs such as AuNPs, AgNPs, and IONPs as anticancer drug delivery systems over the years. For example, Mukherjee et al. [161] reported on the ability of *Eclipta Alba* leaf-synthesized AuNPs to effectively deliver doxorubicin to breast cancer cells (MCF-7 and MDA-MB-231). Later, the same researchers further reported on the ability of *Peltophorum pterocarpum*-synthesized AuNPs to deliver doxorubicin to lung and melanoma cancer cells (A549 and B16F10, respectively) [162]. Pooja and co-workers [163] reported on the ability of gum karaya-stabilized AuNPs to effectively deliver an anticancer drug, gemcitabine hydrochloride, to A549 cells. In another study, Patra and colleagues [89] demonstrated the ability of *Butea monosperma*-synthesized AuNPs and AgNPs to deliver doxorubicin to B16F10 and MCF-7 cancer cells; the anticancer activity of the NPs-based drug delivery systems (DDSs) showed more significant cancer cell inhibition compared to that of the free doxorubicin drug. Furthermore, Firdhouse and Lalitha [164] also reported on the ability of the *Alternanthera sessilis*-synthesized AgNPs to deliver cisplatin to MCF-7 cells. The anticancer activity of the nanoparticle DDS was shown to be superior to the free anticancer drug, cisplatin. Gul et al. [165] reported on the ability of starch-coated *Poa annua*-synthesized AgNPs to deliver an anticancer drug (*Euphorbia dracunculoides* Lam. Plant extract) (AgNPs-EDL@Starch) to SCC7 cancer cells. The AgNPs-EDL@Starch demonstrated a significant anticancer effect on the tested cancer cells. Oladipo and co-workers [166] fabricated hydrophilic bimetallic gold/platinum (AuPt) NPs using a *Phragmites australis* leaves aqueous extract and demonstrated their ability to efficiently deliver doxorubicin to breast and lung cancer cells. Recently, Zadeh and co-workers [167] reported on the ability of *Mentha piperita*-formulated Fe_2_O_3_ NPs to deliver doxorubicin in MCF-7 cells; the nanoparticle DDS was shown to more significantly inhibit the tested cancer cells compared to that of the free anticancer drug. A limited number of drug delivery studies involving bimetallic NPs has been reported, thus there is still scope for the investigation of new combinations. Overall, it can be concluded that the green-synthesized metal NPs can be attractive, non-toxic, and eco-friendly candidates for drug delivery or nanomedicine applications. Taken together, major research endeavours in the areas of cancer diagnosis, imaging, drug delivery and therapeutics have been made over the years. With growing advancements in nanotechnology and the practical application of metal NPs as sophisticated therapeutics or drug/gene delivery modalities, it is only a matter of time before there is a breakthrough in the treatment of cancer.

## 5. Metal Nanoparticles as Novel Antivirus Therapy: Effect against Various Viruses

Viruses are microscopic entities that are composed of a genome/genetic material (RNA or DNA) core, which is coated by a protein layer or a fatty envelope layer. These are pathogens that cause diseases such as the common cold, Flu/Influenza, Respiratory syncytial virus (RSV), Ebola, Human immunodeficiency virus (HIV), Chickenpox, SARS-CoV-2, and others. Viruses such as RSV, HIV, Influenza, Hepatitis, Ebola, and SARS-CoV-2 are enveloped viruses with single-stranded ribonucleic acid (ssRNA) genomes, while Herpes simplex viruses (HSV) are enveloped, double-stranded DNA viruses [31]. Viruses infect living, normal cells by transferring their genome into the host cells and taking over the host cell’s replication mechanism to produce more viruses. Different viruses affect different cells in the body, such as the cells of the blood, respiratory system, liver, reproduction, gastrointestinal system, brain, and skin. Viruses can be transferred and spread through the air, saliva, touch, sexual contact, food or water contamination, and insect vectors. Metal NPs have played a huge role in the management and treatment of a range of viral infections due to their antiviral activity and ability to bind with biomaterials coats (e.g., viral protein coats), which inactivates the virus or blocks the entry of the virus into the host cells by binding to the cells’ surface receptors [31,168]. The nano-size of metal NPs allows them to cross over cell membranes and inhibit the replication of the virus [6]. Lastly, due to their ability to target various regions of the virus, they exhibit limited resistance when they are applied repeatedly [169]. Amongst a range of metal NPs, AgNPs and AuNPs have demonstrated their antiviral effect against a broad range of viruses (Table 5).

Silver NPs as Antiviral Agents: The antiviral activity of both chemical and green-synthesized AgNPs has been previously reported against viruses such as Sunthemp rosette virus (SHRV) [170], Yellow mosaic virus (BYMV) [171], Tomato mosaic virus (ToMV) [172], Potato virus Y (PVY) [172], Dengue virus [173], Feline coronavirus (FCoV) [174]; HIV [175], Influenza A virus [176,177], Chikungunya virus [178,179], Human parainfluenza virus type 3 [180], Bovine herpesvirus [181], Herpes simplex virus-2 (HSV-2) [182], and Norovirus [183], HSV-1 [184], Hepatitis A virus-10 (HAV-10) virus [185], Hepatitis virus, Coxsackie B4 virus [186], RSV [168], and the Newcastle viral disease (NDV) [187]. This antiviral activity is said to be due to the ability of the AgNPs to inhibit the replication of the virus by binding to the viral glycoproteins (gp120) and blocking the virus penetration into the host cell [188], as demonstrated in a case of HIV-1 virus study [175].

For instance, Sujitha et al. [173] synthesized AgNPs using *Moringa oleifera* seed extract and tested their antiviral activity against Dengue virus (DEN-2); they found out that the NPs were spherical and were 100 nm in size and showed significant antiviral activity at a dose of 10.24–21.17 ppm in C6/36 and Vero viral models. Fatima et al. [176] fabricated AgNPs using *Cinnamomum cassia* H7N3 and tested their antiviral activity against Influenza A Virus; they reported that the NPs were spherical, had a range of 25–55 nm in size, and showed great antiviral activity in Vero cells at a dose of 125 μg/mL. In another study, AgNPs were synthesized using *Curcumin* extract, and their antiviral activity was tested against RSV; it was reported that the NPs were spherical, had a range of 11–12 nm in average size, and exhibited good antiviral activity on the infected Hep-2 infected cells at inhibitory concentrations range between 0.008–0.12 nM. The antiviral activity was said achieved to be via the reduction of cytopathic effects, which led to the inactivation of the virus before entry into the host cell [168]. In 2018, Sreekanth and co-workers [177] formulated AgNPs using Ginseng root extract and studied their antiviral activity against Influenza A virus; the NPs were spherical, had a range of 2–50 nm, and showed antiviral activity against MDCK cells at a dose range of between 0.02 and 0.25 M. Sharma and colleagues [179] synthesized AgNPs using *Andrographis paniculate* (AP-AgNPs)*, Phyllanthus niruri* (PN-AgNPs)*, Tinospora cordifolia* (TC-AgNPs) and tested their antiviral activity against Chikungunya virus; they reported that the NPs were spherical, had ranges of 70–95 nm (AP-AgNPs), 70–120 nm (PNAgNPs), 50–70 nm (TCAgNPs), and showed significant antiviral activity in Vero cells at doses of 31.25 μg/mL (AP-AgNPs), 125 μg/mL (PN-AgNPs), and 250 μg/mL (TC-AgNPs). In another study, AgNPs were synthesized using *Lampranthus coccineus* and *Malephora lutea* plant extracts, and their antiviral activity was tested against HSV-1, HAV-10 virus, and Coxsackie B4 virus; it was observed that the NPs were spherical, had a range of 10.12–27.89 nm in size, and showed great antiviral activity against Vero cells at a dose of 5.13 μg/mL [186]. Other studies reported on AgNPs synthesized using seaweed to treat HSV-1 and HSV-2; it was demonstrated that the NPs were spherical, had a range of 8–27 nm in size, and showed significant antiviral activity against Vero cells at a dose of ID50-2.5 μL [184,185].

Gold NPs as Antiviral Agents: Several studies have been previously reported regarding the antiviral activity of AuNPs or functionalized AuNPs against viruses such as HIV, HSV-1, Herpes, Hepatitis, adenovirus, West Nile virus, Influenza, and Measles virus (MeV) over the years. For instance, Bowman et al. [189] synthesized AgNPs conjugated with an HIV inhibitor, TAK-779, and evaluated them against HIV infection. The CCR5-tropic HIV-1 clone JR-CSF-infected phytohemagglutinin-stimulated peripheral blood mononuclear (PBM) cells were treated with the TAK-779 conjugate. Post-treatment, it was observed that on its own, the inhibitor demonstrated no antiviral activity, while when it was conjugated onto the AuNPs, it showed anti-HIV activity with an IC_50_ of 10 nM through the inhibition of the viral replication. The findings suggest that the conjugation of an inactive drug/inhibitor onto AuNPs could activate/enhance the therapeutic effect of the drug/inhibitor. Di Gianvince et al. [190] demonstrated the ability of AuNPs to interact with the HIVs envelop glycoprotein and prevent virus replication. Other functionalized AuNPs that have demonstrated significant anti-HIV effects include AuNPs conjugated with carbohydrate and inhibitors lamivudine/abacavir [191], AuNPs coated with raltegravir [192], AuNPs conjugated with amino acid L-Cysteine [193], AuNPs conjugated with polyethylene glycol [194], and AuNPs conjugated with peptide triazoles [195]. It has been reported that the anti-HIV activity of these NPs is facilitated by their charged surface that can interact with the cationic amino acids, which are saturated on the viral envelope glycoprotein gp120, thus blocking the replication of reverse transcriptase enzymes [193]. Furthermore, Baram-Pinto et al. [196] demonstrated the antiviral activity of AuNPs coated with mercarptoethene sulfonate against the HSV-1 virus. The NPs were non-toxic and exhibited the anti-HSV-1 by inhibiting the binding of the virus to the cell, thus changing the vulnerability of the cell to the virus. In a recent study, the anti-HSV-1 virus effect of safe, eco-friendly, and non-toxic bare AuNPs synthesized using *Spirulina platensis* was demonstrated [197]. In the same year, Lee et al. [198] reported on the antiviral activity of AuNPs coated with hyaluronic acid and interferon for hepatitis C treatment, while the effect of bare AuNPs on Hepatitis C has also been reported [199]. In 2013, Niikura et al. [200] demonstrated the anti-West Nile activity of AuNPs coated with West Nile virus envelope protein in an *in vivo* study using mice. In another study, Feng et al. [201] demonstrated the anti-Influenza activity of AuNPs conjugated with thiosialoside molecules. Later, Lysenko et al. [202] demonstrated the anti-adenovirus effect of AuNPs coated with silicon dioxide and AuNPs coated with silicon. Both NPs significant antiviral activities of 100% and 96%, respectively. Melendez-Villanueva et al. [203] also showed the antiviral activity of AuNPs prepared using garlic extract against the MeV virus.

Copper NPs as Antiviral Agents: The antiviral activity of copper compounds and copper oxide has been demonstrated against several viruses. In 2015, Hang and co-workers [204] demonstrated the ability of copper oxide NPs to affect hepatitis C virus by inhibiting by blocking the virus attachment and entry stages. Other studies have demonstrated the antiviral activity of copper compounds against several other viruses such as influenza, norovirus, and coronaviridae [205,206,207,208,209], and a general review on this was reported by Cortes and Zuñiga [210]. Yugandhar and co-workers [211] reported on the antiviral activity of CuO NPs prepared using *Syzygium alternifolium* fruit extract against Newcastle disease virus (NDV); a limited about of literature is available regarding the green-synthesized approach of these NPs, hence more research is encouraged. The antiviral activity of these NPs can be ascribed to the controlled release of copper ions which results in oxidative stress activation; these NPs also destroy and disrupt the genome and capsid of the virus [212,213].

Other Metal NPs as Antiviral Agents: The antiviral activities of other metal NPs such as iron oxide/ferromagnetic NPs (IONPs, Fe_3_O_4_ NPs)/superparamagnetic iron oxide NPs (SPIONPs), silica NPs (SiNPs), zinc oxide NPs (ZnO NPs), cerium dioxide NPs (CeO_2_ NPs), and titanium oxide NPs (TiONPs) have been demonstrated against various viruses evaluated in several studies [31,214,215,216]. Fe_3_O_4_ NPs exhibited antiviral activity against various types of influenza virus [217], while most IONPs can act as diagnostic agents [218,219], and functionalized SPIONPs can act as antiviral drug delivery agents [220]. SiNPs and functionalized SiNPs exhibited antiviral activity against HIV, human papillomavirus, HBV [221], influenza A virus [222], and HSV-1 and HSV-2 [223]. These NPs could also be used as detection/diagnostic agents [224]. ZnO NPs and functionalized ZnO NPs exhibited antiviral activity against the H1N1 influenza virus [225]. Green-synthesized ZnO NPs exhibited a healing/antiviral activity against TMV [226]. CeO_2_ and functionalized CeO_2_ demonstrated an antioxidative activity with low cytotoxicity [227], enhancing the bioactivity of interferon (IFN), one of the constituents of the influenza vaccines, and enhance vaccine efficiency [228,229]. Thus, further research is needed to study the effectiveness of CeO_2_ optimized vaccines against SARS-COV-2. TiNPs exhibited an antiviral activity against influenza viruses (H3N2, H5N1, H1N1, and H9N2) [230,231]. To the best of our knowledge, no studies have been reported on the antiviral activity of bimetallic NPs against viruses. Limited literature report on their anticancer and antibacterial activities, thus much research is still needed to explore their potential as antiviral agents.

Therefore, from this catalogue of the previously reported studies, it can be highlighted that the green-synthesized metal NPs present safe, eco-friendly, cheap, and more accessible antiviral therapeutics, and these could be beneficial in the cases of viral outbreaks which require urgent attention. Furthermore, the studies also highlight the beneficial effect of coupling the antiviral potential of NPs with the antioxidant, antiviral, anti-inflammatory activities of bioactive compounds, such as flavonoids, or antiviral drugs, such as remdesivir and chloroquine, which could be the next step towards COVID-19 eradication.

**Table 5:** Antiviral activity of metal NPs against various viruses and possible mechanisms.

Metal	Synthesis Material/Conjugate	Physicochemical Properties	Virus	Antiviral Activity	Mechanism of Action	References
Silver	Biological (*Moringa oleifera* seed extract)	Spherical shape; 100 nm mean diameter size	Dengue virus	*In vitro*: C6/36 and Vero infected cells; IC_50_ value range of 10.24–21.17 ppm	Not available	[173]
Silver	Biological (*Cinnamomum cassia* H7N3)	Spherical shape; 25–55 nm diameter size range	Influenza A virus	*In vitro*: Vero infected cells; IC_50_ value of 125 μg/mL	Not available	[176]
Silver	Biological (*Curcumin* extract)	Spherical shape; 11–12 nm diameter size range	Respiratory syncytial virus	*In vitro*: Hep-2 infected cells; IC_50_ value of 0.008–0.12 nM	Reduction of cytopathic effects which led to the inactivation of the virus before entry into the host cell	[168]
Silver	Biological (Ginseng root extract)	Spherical shape; 2–50 nm diameter size range	Influenza A virus	*In vitro*: MDCK infected cells; IC_50_ value range of 0.02–0.25 M	Not available	[177]
Silver	Biological (*Andrographis paniculate; Phyllanthus niruri; Tinospora cordifolia* extracts)	Spherical shape; 70–95 nm (AP-AgNPs), 70–120 nm (PNAgNPs), 50–70 nm (TCAgNPs) diameter size range	Chikungunya virus	*In vitro*: Vero infected cells; IC_50_ values of 31.25 μg/mL (AP-AgNPs); 125 μg/mL (PN-AgNPs); 250 μg/mL (TC-AgNPs)	Not available	[179]
Silver	Biological (*Lampranthus coccineus* and *Malephora lutea* extracts)	Spherical shape; 10.12–27.89 nm diameter size range	Herpes simplex virus-1, Hepatitis A virus-10, and Coxsackie B4 virus	*In vitro*: Vero infected cells; IC_50_ value of 5.13 μg/mL	Not available	[186]
Silver	Biological (seaweed)	Spherical shape; 8–27 nm diameter size range	Herpes simplex virus-1 and 2	*In vitro*: Vero infected cells; IC_50_ value of ID50–2.5 μL	Not available	[184]
Gold	Chemical (AgNPs coated with 100% and 50% density of the sulfated ligand)	Spherical shape; 1.7–2.6 nm diameter size range	Human immunodeficiency virus	*In vitro*: MT-2 infected cells; IC_50_ value of 1.29 and 2.32 μg/mL	Interaction with the HIVs envelop glycoprotein and prevent virus replication	[190]
Gold	Chemical (AuNPs coated with hyaluronic acid/interferon α)	Spherical shape; 46.03 nm diameter size range	Hepatitis C virus	*In vitro*: Daudi infected cells; IC_50_ not available	Enhancement of the expression of 2′, 5′- oligoadenylate synthetase 1 enzyme which activates innate immune responses to viral infection	[199]
Gold	Chemical (AuNPs conjugated with an HIV inhibitor, TAK-779)	Shape not available; 2.0 nm diameter size	Human immunodeficiency virus	*In vitro:* PBM infected cells; IC_50_ value of 10 nM	Inhibition of the viral replication	[189]
Gold	Chemical (AuNPs conjugated with carbohydrate and inhibitors lamivudine/abacavi)	Spherical, shape; 3 nm diameter size	Human immunodeficiency virus	*In vitro*: TZM-bl cells; IC_50_ value of 1 µM and 8 µM	Interaction with the cationic amino acids on the viral envelope glycoprotein gp120, thus blocking the replication of reverse transcriptase enzymes which prevents viral replication	[191]
Gold	Chemical (AuNPs coated with mercarptoethene sulfonate)	Spherical shape; 4 nm diameter size	Herpes simplex virus-1	*In vitro*: VeroE6 infected cells; IC_50_ not available	Inhibition of viral attachment, entry, and cell-to-cell spread	[196]
Gold	Biological (*Spirulina platensis* extract)	Octahedral, pentagonal, and triangular shapes; 15.60–77.13 nm diameter size range	Herpes simplex virus-1	*In vitro*: VeroE6 infected cells; IC_50_ value not available (90% cytopathic effect at 31.25 μL)	Inhibition of the viral replication	[197]
Gold	Chemical (AuNPs coated with hyaluronic acid and interferon)	Spherical shape; 29.16 nm diameter size	Hepatitis C virus	*In vitro*: Daudi infected cells; IC_50_ not available	Enhancement of the expression of 2’-5’ oligoadenylate synthetase 1 enzyme which activates innate immune responses to viral infection	[198]
Gold	Chemical (AuNPs coated with West Nile virus envelop protein)	Non-spherical, rod, and cube shapes 20–40 nm diameter size range	West Nile virus	*In vitro*: BMDCs; IC_50_ value of not available	Induction of IL-1β, IL-18, TNF-α, IL-6, IL-12 antibodies, and granulocyte– macrophage colony-stimulating factor	[200]
Gold	Chemical (AuNPs conjugated with thiosialoside)	Spherical shape; 5 nm and 20 nm diameter size	Influenza virus	*In vitro*: Chicken red blood cells (CRBCs); IC_50_ value not available	Inhibition of haemagglutin-in	[201]
Gold	Chemical (AuNPs coated with silicon dioxide; silicon)	Spherical shape; 5 nm and 100 nm diameter sizes	Adenovirus	*In vitro*: MDBK (the Madin-Darby bovine kidney line) and Hep-2 cells; IC_50_ value not available (66–86% virucidal effect)	Inhibition of virus reproduction due to field effects	[202]
Gold	Biological (garlic extract)	Spherical shape; 11 nm diameter size	MeV virus	*In vitro*: Vero infected cells; EC_50_ value of 8.829 µg/mL	Blockage of viral receptors resulting in a significant reduction of viral infection	[203]
Copper oxide	Chemical (CuONPs coated with cetyltrimethylammonium bromide)	Spherical shape; 45 nm diameter size	Hepatitis C virus	*In vitro*: Huh7 infected cells; IC_50_ value not available	Blockage of virus during the attachment and entry stages	[204]
Copper oxide	Biological (*Syzygium alternifolium* fruit extract)	Spherical shape; 2–69 nm diameter size	Newcastle disease virus	*In ovo*: Infected eggs; The embryo infectious dose (EID_50_) value was 10^6.5^/mL and EC_50_ value of 50.98 μg/mL	Activation of oxidative stress; disruption of the genome and capsid of the virus	[211]

### 5.1. Antiviral Properties of Metal Nanoparticles against COVID 19/SARS-CoV-2 and Possible Mechanisms of Action

**Figure 3:**
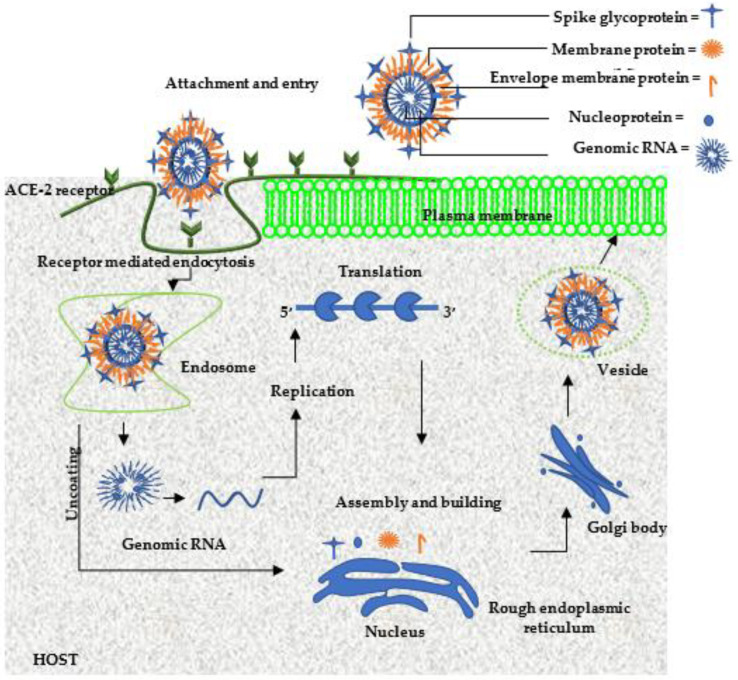
The life cycle of COVID-19/SARS-CoV-2.

Coronavirus, COVID-19, caused by SARS-CoV-2, has emerged as one of the fastest spreading and most lethal infectious diseases, claiming millions of lives worldwide [8]. This is an ssRNA-enveloped nanometer (120–160 nm) virus that belongs to the subfamily of Orthocoronavirinae, the family of Coronaviridae, the order of Nidovirales, and the realm of Riboviria [232,233]. This virus comprises four genera, namely, alpha (α), which has been reported to infect humans; beta (β), which has been reported to infect humans; gamma (γ), which has been reported to infect birds; delta (δ), which has been reported to infect birds [234,235]. It is composed of structural and non-structural proteins such as spike, helicase, phosphorylated nucleocapsid, envelope, papain-like protease, 3-chymotrypsin-like protease, and membrane glycoproteins [9]. The SARS-Co-V-2 infection process, as illustrated in Figure 3, starts with the attachment of the virus to the human host cell angiotensin-converting enzyme-2 (ACE-2) receptors, followed by entry into the host cell via receptor-mediated endocytosis, then the accumulation of viral load into endosome vesicles; the viral genomic RNA is then released into the cytosol for processing to form replicative templates, and then it enters the nucleus of the host cell; this is then replicated, transcribed, and translated. The translated viral genomic RNA then goes through reassembly, and is then released as new virions. These new virions quickly bind to the next host cell, and the process of infection and replication is repeated [236]. Conventional antiviral drugs in clinical trials such as remdesivir and chloroquine are inadequate in treating this disease as they induce side effects such as increasing the hepatic enzyme levels, gastrointestinal effects, and cardiotoxic effects [13,237]. Therefore, new strategies that are effective, site specific, and safe are urgently needed to treat this virus. Recently, several research have centred around the incorporation of metal NPs as either diagnostics, therapeutics, or antiviral delivery nanosystems to try to combat this disease [23]. This can be attributed to the various unique properties of metal NPs, as well as their ability to target various sites of the life cycle of SARS-Co-V-2 [22,23], as illustrated in Figure 3.

**Figure 4:**
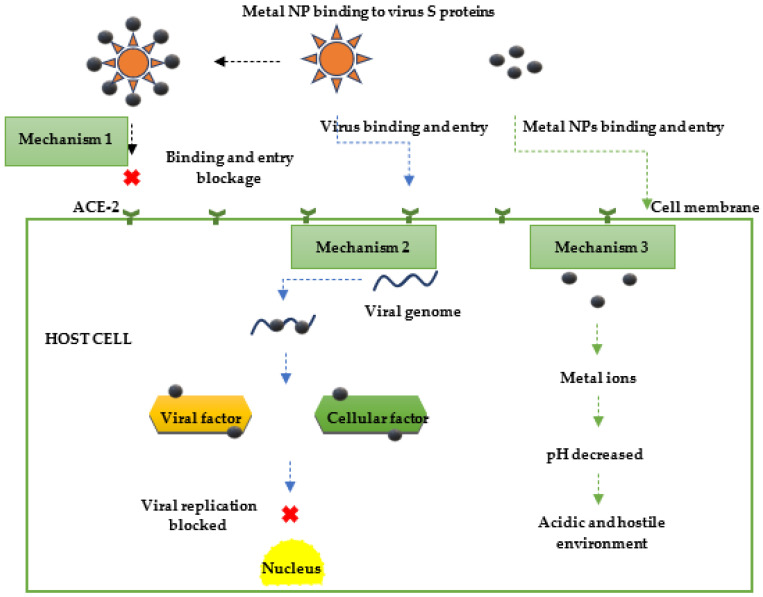
Possible antiviral mechanisms of metal nanoparticles.

The antiviral effect and efficacy of metal NPs depend on their antioxidant and physicochemical features such as metal oxides, morphology, and size. Functionalized metal NPs interact with host cells and viruses more than bare/non-functionalized NPs do [238]. Regarding the antiviral mechanism of action, different hypotheses have been postulated concerning the exact sequential mechanism of metal NPs and virus interactions (Figure 4). Firstly, the richly charged surface of the metal NPs enables them to bind to the virus surface, thus directly inactivating the virus or hindering the entry of the virus into the host cell by interacting with the overexpressed, non-conserved ACE-2 human host cell surface receptors [168]. Further, it has been postulated that the nano-scale size and the high surface area–volume ratio of metal NPs allows them to bind and penetrate the host cells and interact with the viral envelope proteins, impeding the replication of the viral genome [239]. It has also been postulated that the antioxidant property of metal NPs allows them to stimulate the ROS generation, which decreases the pH of the airway epithelium, making the environment become more acidic and difficult for a virus to survive by acting as ion tanks for the controlled release of ions from bioactive molecules [6]. The metal ions generated are also capable of binding and inhibiting the virus’s respiratory enzymes and interfering with the nucleic acid; this has been previously shown against a range of viruses [23,240]. Lastly, it has been reported that the infected cell components are generally saturated with host and viral cellular factors that encode for viral replication and progeny virions production. Thus, it has been said that the interaction of these factors and metal NPs could also be another antiviral mechanism of action [169].

The development of a novel, effective anti-COVID-19/SARS-CoV-2 treatment remains a challenge to date, thus much relevant research is ongoing. For now, the consensus is that essential anti-SARS-CoV-2 therapeutics and drug designing, discovery, and development can be achieved through (a) targeting key components of the SARS-Co-V-2 life cycle, including the S protein, the protease/M Pro/3CL pro, the helicase, and the reverse transcriptase inhibitors, the RNA polymerase, and the 15 endoribonucleases [241]; (b) the designing of nanomaterials that are capable of conjugating with FDA approved antiviral drugs [242]. In this case, the role of the cost-effective nanocarrier is to enhance the drug efficacy by providing protection to the drug from degrading enzymes, enhancing targeted drug release, increasing the bioavailability of the drug, and reducing/eradicating any side effects [243]. Functionalizing the surface of this nanocarrier with fitted moieties/linkers enhances the ability of the antiviral drug to bind with the virus, thus enhancing the bioavailability of the drug in the virus. The use of metal NPs as drug delivery platforms is also dependent on the advancement of antiviral drugs that can target the SARS-CoV-2 life cycle through the target site of action, as previously mentioned [244].

Interestingly, several studies have demonstrated the efficacy of some metal NPs against COVID-19/SARS-CoV-2 (Table 6) through the inhibition of structural targets of SARS-Co-V-2 that are associated with virus replication and mutation [245]. For instance, in patients infected with COVID-19, a significant increase in the levels of proinflammatory cytokines; interleukins (IL)-2, IL-7, IL-10; tumour necrosis factor-alpha (TNF-α); interferon-inducible protein 10 (IP-10); macrophage incendiary protein-1A (MIP-1A); granulocyte-colony-stimulating factor was observed upon treatment with certain metal NPs [246,247]. Thus, modulating the inflammatory cytokines could be an approach to reduce the infection rate of COVID-19. Recently, metal NPs such as AgNPs and AuNPs have been shown to exhibit significant antiviral effects against SARS-Co-V-2 due to their ability to neutralize the viral infection by enhancing the generation of key anti-inflammation and anti-cytokine factors [248]. AuNPs, in particular, have been demonstrated to possess the ability to enhance the inflammatory response by increasing the expression of IL-6, IL-1 beta, and interferon-gamma (INFY-γ) [249,250]. Also, the great biocompatibility of AuNPs makes them good candidates to produce COVID-19 vaccines as they can easily be dispersed intranasally, as well as via the lymph nodes, and they can enhance the immune system by activating CD8^+^ [251]. AuNPs can also neutralize the viral infection by binding to the S protein of SARS-Co-V2 and blocking the viral entry into the host cells, and this was reported in an *in vivo* study reported by Sekimukai and co-workers [153]. Herein, the researchers reported on the ability of AuNPs to act as a vaccine adjuvant against SARS-CoV-2. In this study, the S protein of the virus was targeted for blocking the virus infection. The NPs were used as both the antigen carrier and as an adjuvant with the subunit vaccines. Then, following treatment of the SARS-CoV-2 infected BALB/c mice with the antigen-conjugated AuNPs, a strong antigen-specific IgG response was exhibited against the infected BALB/c mice [153]. In the same year, Rothan et al. [29] reported on the antiviral activity of an FDA approved gold-conjugated-triethyl phosphine drug (used for treatment of rheumatoid arthritis), Auronafin, against SARS-CoV-2 infection. The antiviral effect of the drug was reported to be achieved via the suppression of SARS-CoV-2-associated cytokines in Huh7-infected human cells with an EC_50_ value of 1.4 μM.

Moreover, Te Velthuis et al. [252] reported on the antiviral activity of zinc conjugated with pyrithione against SARS-CoV-2 infection. The demonstrated antiviral activity was said to be achieved via the inhibition of replication by hindering the virus’s RNA polymerase of the multiprotein replication and transcription complexes. Thus, other recent application suggestions include combining zinc with drugs such as azithromycin (antibiotic), hydroxychloroquine, or chloroquine for the efficient treatment of COVID-19 [253]. Recently, several molecular docking studies have demonstrated the ability of metal NPs to bind with some target regions. For example, Abo-Zeid and co-workers [254] showed the ability of IONPs (Fe_2_O_3_ and Fe_3_O_4_ NPs) to effectively bind with the S protein receptor-binding domain (S-RBD) of SARS-CoV-2. Further, the antiviral activity of graphene nanomaterials has also been reported, and this effect was ascribed to their large surface area that can form conjugates with sulphated antiviral drugs, which enables easier interaction with the virions residues, resulting in the blocking of virus replication [255]. Sulphated heparin is an antiviral drug that can bind with the S protein of SARS-CoV-2; thus, graphene nanomaterials can be a platform for such drug, and hence, synergistically, this sulphated heparin functionalized graphene platform can contribute greatly as a therapeutic against COVID-19/SARS-CoV-2 virus [256].

Several studies have been reported on the antiviral activity of either chemical or green-synthesized AgNPs against SARS-Cov-2 over the past two years, thus much research is still needed to fully explore these NPs, more so, the green-synthesized nanoparticles, as they could provide a safer and cheaper alternative. Jeremiah et al. [257] investigated the antiviral activity of AgNPs against Vero cells infected with the SARS-Cov-2 virus. They found out that the NPs demonstrated a significant antiviral activity on the infected cells, with IC_50_ values between 1 and 10 ppm. The antiviral activity was reported to be caused by the inhibition of the viral entry and the disruption of viral integrity. In another study, Medhi and co-workers [258] reported on the antiviral activity of capped AgNPs; they observed that the NPs inhibited the synthesis of the negative RNA of the porcine epidemic diarrhoea virus (PEDV), an alphacoronavirus (alpha-CoV). The antiviral activity was said to be caused by the suppression of the innate immune response, eradicating any chance of viral mutation. Almanza-Reyes and co-workers [259] reported on the antiviral activity of AgNPs against SARS-CoV-2-infected cells. They observed that the AgNPs demonstrated an antiviral activity of about 80% on the tested cells at an inhibitory concentration of 0.03%. In another study, Al-Sanea et al. [260] synthesized AgNPs using strawberry (*Fragaria ananassa* Duch.) and ginger (*Zingiber officinale*) methanolic extracts to study their SARS-CoV-2 antiviral potential. The NPs were spherical with average sizes of 5.89 nm and 5.77 nm, respectively. Both the strawberry- and ginger-mediated AgNPs demonstrated significant antiviral activity against SARS-CoV-2. The antiviral activity was credited to the binding of various compounds of the extracts, neohesperidin to be specific, with both the SARVS-2 NSP16 viral protein and human AAK1 host protein as predicted by the dynamics and molecular docking simulation studies. The researchers then suggested that future studies could include developing these green NPs into novel anti-SARS-CoV-2 drugs [260].

**Table 6:** Antiviral activity of metal NPs and their functionalized counterparts against COVID 19/SARS-CoV-2 and possible mechanisms of action.

Metal	Conjugate/Adjuvant	Size	Antiviral Activity	Mechanism of Action	References
Gold	SARS-CoV S protein 40	40 nm and 100 nm	*In vivo*: infected BALB/c mice; IC_50_ value not available	Induction of strong antigen-specific IgG response	[153]
Gold	Triethyl phosphine (Drug, auronafin)	Not available	*In vitro*: Huh7 infected human cells; EC_50_ value of 1.4 μM	Inhibition of viral replication by suppressing virus associated cytokines	[29]
Zinc	Pyrithione	Not available	*In vitro*: VeroE6 infected cells; IC_50_ value of 1.4 mM	Inhibition of replication by hindering the virus’s RNA polymerase of the multiprotein replication and transcription complexes	[252]
Iron oxide (Fe_2_O_3_ and Fe_3_O_4_)	-	Not applicable	Not applicable	Binding with the S protein receptor-binding domain (S-RBD)	[254]
Silver	-	Around 10 nm	*In vitro*: VeroE6 and d Calu-3 infected cells; IC_50_ value not available	Inhibition of the viral entry, and disruption of viral integrity	[257]
Silver	Strawberry (*Fragaria ananassa* Duch) and ginger (*Zingiber officinale*); methanolic extracts	5.89 nm and 5.77 nm	*In vitro*: Vero infected cells; IC_50_ of values of 0.0062 µg/mL (ginger extract) and 0.0989 µg/mL (strawberry extract)	Binding of various compounds of the extracts, neohesperidin to be specific, with both the SARVS-2 NSP16 viral protein and human AAK1 host protein	[260]

### 5.2. Applications of Metal Nanoparticles against COVID-19/SARS-CoV-2

**Figure 5:**
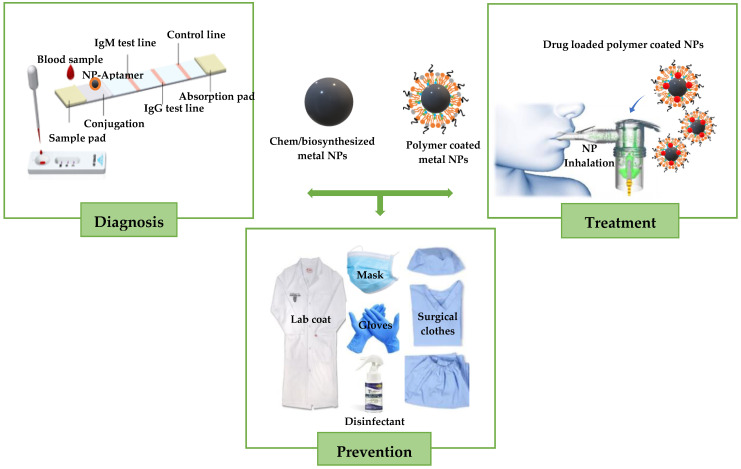
Schematic illustrations of the applications of metal nanoparticles against COVID-19/SARS-CoV-2.

Metal NPs have been successfully applied in the treatment of various viral diseases over the years [169], and have recently showed potential applicability in several diagnostic, preventative, and treatment endeavours during the COVID-19/SARS-CoV-2 pandemic (Figure 5). In 2020, international organisations such as Massachusetts Institute of Technology (MIT) and World Nano Foundation (WNF) reported on the possible applicability or implementation of AuNPs for the development of new fast detection techniques for COVID-19, such as the “IgM/IgG antibody assay kit” and AuNPs-based strips. These strips were said to be coated with antibodies (Abs) that will bind with the SARS-COV-2 (viral antigen), while the other Abs were to be linked with AuNPs. It was postulated that the interaction of the strip (which contains Abs) with a sample of either urine or blood (which contains viral antigens) will cause a colour change, indicating a positive COVID-19 test [261,262]. In the same year, it was reported that a solution of Ag^+^ ions and TiO_2_ was used to disinfect the streets in Milan (Italy) (Nanotech Surface Coronavirus 2020). Recently, Kumar et al. [263] fabricated a durable and facile fabric containing photo-deposited AgNPs for use in the fight against SARS-CoV. The AgNPs on the cloth were approximately 10 nm in size and demonstrated a 97% antiviral activity against SARS-CoV-2, which was mediated by the redox Ag^+^ ions formed during the production of the AgNPs-coated cloth [263]. Other medicinal clothing applications of AgNPs are discussed in detail in a review in [264]. Furthermore, Takeda and co-workers [265] evaluated the antiviral activity of copper iodide (CuI) NPs colloids, CuI-doped fabric, and CuI-doped film against SARS-CoV-2. They observed that the CuI colloids displayed time-dependent antiviral activity, which was via the ROS species generation and destruction of viral proteins. In the case of the CuI-doped fabric and film, the antiviral activity that was demonstrated indicated that both these could be used as anti-SARS-CoV-2 protective materials such as surgical masks/protective clothes/gloves [265]. Sarkar [266] reported on the possible applicability of inhaled AgNPs as a novel anti-COVID-19 therapy. The author hypothesized that the administration of low doses of colloidal AgNPs (with a diameter size of 10 nm) conjugated with bronchodilators in the lungs of COVID-19 patients through a nebulizer (converts medication from a liquid form to a mist form) could treat the viral infection. It was postulated that once the misty AgNPs-tagged-bronchodilators were inhaled into the lung cells, the AgNPs will convert into Ag^+^ ions, which will then bind to the sulphur or phosphorus biomolecules of the virus, thus inactivating the virus [266]. This proof-of-concept was evaluated and validated to be true as an effective novel therapy for prevention and treatment of COVID-19 infections at early stages [267]. Further developments of these NPs through surface functionalization with biocompatible polymers, targeting moieties and FDA approved antiviral drugs, could synergistically contribute greatly to the overall therapeutic effect against COVID-19/SARS-CoV-2 virus, which could be the next step towards eradication of this disease.

## 6. Limitations of Metal Nanoparticles in Clinical Applications

The successful application of metal NPs as nanotherapeutics is limited by factors such as nanotoxicity, method of preparation, biodegradability, and clearance [268]. Nanotoxicity is caused by non-specific target effects of metal NPs, which results in the activation of unwanted immunogenicity, inflammation mediators, autoimmune responses, and allergy responses [269,270]. The non-specific target effect of bare metal NPs is associated with their surface reactivity, size, and morphological features [271]. It has been reported that the various functional groups present on the surface of the metal NPs control the toxicity of NPs. For instance, functional groups such as amines (-NH_2_) and hydroxyl/alcohols (-OH) are more toxic than functional groups such as carboxyl groups (-COOH) are [272]. Furthermore, according to a study by Karlsson et al. [273] metal oxide nanoparticles, such as CuO NPs, IONPs, and TiO_2_ NPs, demonstrated more toxicity compared to that of their microparticle counterparts when they were tested against the A549 cell line [273]. This increased toxicity was due to the oxidative activity possessed by these metal oxide NPs, as well as the enhanced cell membrane permeability of the NPs (due to nano sizes) and increased systemic bioavailability which, lead to increased intracellular constituent dysfunction and damage compared to those of their counterparts [274]. Several solutions have been proposed to reduce the nanotoxicity of metal NPs. These include (a) using a safer synthesis approach; (b) modification of the surface of the NPs using biocompatible and biodegradable capping/stabilizing agents (e.g., chitosan, poly-lactic-co-glycolic acid, polyethylene glycol) to control the size, stability [275,276], and enhance reactivity for effective binding to the cell surface and other drugs; (c) understanding the dynamics involved in the interactions between biological systems (cells or viruses) and NPs; (d) evaluating the *in vivo* toxicity and safety concerns [277]. Moreover, the biodegradability of chemically synthesized metal NPs is either non-existent or slow, which poses serious toxicity concerns [278]. These NPs are usually either non-degradable or contain some non-degradable constituents, which tend to be retained in the system and accumulate in vital tissues/organs, causing systemic toxicity/nanotoxicity [31,230]. Sometimes, even the degraded constituents of the biocompatible chemically produced metal NPs may lead to nanotoxicity [279]. The biodegradability and clearance mechanisms of metal NPs are still not clearly understood, but it has been postulated that the NPs are gradually excreted from the body within 14 days via urine and faeces [280,281]. The use of green synthesis has been proposed as an ideal approach to combat these issues as it produces eco-friendly, biodegradable, and safe metal NPs. Moreover, the coating of the richly charged surfaces of these biosynthesized metal NPs with natural biomolecules such as polymers, polysaccharides, polyethylene glycol derivatives, lipids, peptides, and vitamins is said to enhance their drug loading/encapsulation, intracellular circulation time, and specific drug delivery capacity [158,159]. This is important for the successful *in vivo* biological application of these metal NPs, particularly in therapy.

## 7. Prospects and Conclusions

Metal NPs have shown a great potential to increase the growth of the pharmaceutical market and expand the health benefits for various diseases including cancer and COVID-19. Yet, the present preclinical and clinical translation research gap for nanomedicines is still huge and challenging. Thus, prospects of metal NPs for successful clinical use require a great consideration of their physicochemical properties, agglomeration, and disease pathophysiology during their design phase. This will allow the researchers to tailor design the NPs with optimal therapeutics for specific disease targeting, as well as aid the metal NPs to overcome various biological barriers. Furthermore, extensive *in vitro* evaluation of the nanotoxicity and therapeutic efficiency of the metal NPs is mandatory prior to preclinical assessment. Following this, *in vivo* pre-clinical assessments of nanotoxicity, safety/toxicology, therapeutic efficiency, pharmacokinetics, and biodistribution are also required. Toxicology studies predict the long-term and short-term toxicity of the NPs, and this can be evaluated by monitoring the absorption, biodistribution, biodegradation, immunostimulatory effect, and excretion of the metal NPs. Therefore, tackling these challenges is pivotal to ensuring the successful clinical application of metal NP-based therapeutics in nanomedicine. Overall, this review article has provided a perspective on the potential applicability of metal NPs and metal oxide NPs for cancer and COVID-19 diagnoses, preventions, and treatments, as well as the issues that are still lingering for their full clinical application. This review article will contribute to the vast literature already available regarding this research area.

## Figures and Tables

**Table 1:** Anticancer activity of various biosynthesized gold nanoparticle.

Plant Source	Physicochemical Properties	*In Vitro* Anticancer Activity	References
1. *Podophyllum hexadrum* L. (Leaf extract, H_2_O)	Spherical shape; 5–35 nm diameter size range	HeLa cells; IC_50_ of 20 μg/mL	[80]
2. *Mentha piperita* (Leaf extract, H_2_O)	Spherical, hexagonal, and triangular shape; 10–300 nm diameter size range	HEK293 cells; IC_50_ value not available	[81]
3. *Meringa oleifa* (Leaf extract, H_2_O)	Spherical and polyhedral shape; 10–20 nm diameter size range	A549 cells and SNO cells; IC_50_ values of 98.46 and 92.01 μg/mL	[82]
4. *Rhus chinesis* (Galls extract, H_2_O)	Spherical and oval shape; 20–40 nm diameter size range	Hep3B cells, MG-63 cells, and MKN-28 cells; IC_50_ value of 150 µg/mL	[83]
5. *Nerium oleander* (Stem bark extract, H_2_O)	Spherical, hexagonal, triangular, rod, and flower-like shape; 10–100 nm diameter size range	MCF-7 cells; IC_50_ value of 74 μg/mL	[84]
6. *Curcuma wenyujin* (Rhizome extract, H_2_O)	Spherical shape; 200 nm mean diameter size	A498 cells and SW156 cells; IC_50_ values of 25 µg/mL and 40 µg/mL	[85]
7. *Retroselinum crispum* (Leaf extract, H_2_O)	Spherical, semi-rod, and flower-like shape; 20–80 nm diameter size range	CO-II cells; IC_50_ value of 84.39 µg/mL	[86]
8. *Polianthes tuberosa* L. (Floral extract, H_2_O)	Spherical, triangular, rod, hexagon, and pentagon shape; 10–100 nm diameter size range	MCF-7 cells; IC_50_ value not available	[87]
9. *Tecoma capensis* (Leaf extract, H_2_O)	Spherical shape; 10–35 nm diameter size range	MCF-7 cells; IC_50_ value of 9.6 µg/mL	[88]

**Table 2:** Anticancer activity of various biosynthesized silver nanoparticles.

Plant Source	Physicochemical Properties	*In Vitro* Anticancer Activity	References
1. *Bauhinia tomentosa* Linn (Leaf extract, H_2_O)	Spherical shape; 16.7 nm mean diameter size	A549 cells; IC_50_ value of 28.125 μg/mL	[109]
2. *Artemisia marschalliana* (Aerial extract, H_2_O)	Spherical shape; 5–50 nm diameter size range	AGS cells; IC_50_ value of 21.05 µg/mL	[110]
3. *Mentha pulegium* (Leaf extracts, H_2_O and MeOH)	Anisotropic shape; 5–50 nm diameter size range	HeLa cells and MCF-7 cells; IC_50_ value of approximately 100 µg/mL	[111]
4. *Rheun Rhabarbarum* Rhubarb (Stem extract, H_2_O)	Spherical shape; 5–50 nm diameter size range	HeLa cells; IC_50_ value of 10 mg/mL	[112]
5. *Cynara scolymus* (Leaf extract, H_2_O)	Spherical shape; 200–223 nm diameter size range	MCF-7 cells; IC_50_ value of 10 μg/mL	[113]
6. *Curcuma longa* and *Zingiber officinale* (Rhizome extracts, H_2_O)	Spherical shape; 42–61 nm diameter size range	HT-29 cells; IC_50_ value of 150.8 µg/mL	[114]
7. *Hypericum Perforatum* L. (Aerial extract, H_2_O)	Spherical shape; 20–50 nm diameter size range	HeLa cells, HepG2 cells, and A549 cells; IC_50_ values of 6.72 μg/mL, 6.88 μg/mL), 6.08 μg/mL	[115]
8. *Cowpea* starch (Seed extract, H_2_O)	Spherical shape; 40–70 nm diameter size range	HEK293 cells, MCF-7 cells, and A549 cells; IC_50_ values of 41.7, 56.3, and 63.8 μg/mL	[42]
9. *Allium cepa* L. (Shallot extract, H_2_O)	Cubic shape; 150–250 nm diameter size range	HT-29 cells and SW620 cells; IC_50_ values not available	[116]

**Table 3:** Anticancer activity of various biosynthesized magnetic nanoparticles.

Plant Source	Physicochemical Properties	*In Vitro* Anticancer Activity	References
1. *Sargassum muticum* (Seaweed extract, H_2_O)	Cubic shape; 18 nm mean diameter size	MCF-7 cells, Jurkat cells, HepG2 cells, and HeLa cells; IC_50_ values of 18.75 μg/mL, 6.40 μg/mL, 23.83 μg/mL, and 12.50 μg/mL	[129]
2. *Psoralea corylifolia* (Seed extract, H_2_O)	Spherical, rod, hexagonal, cubic, crystalline shape; 39 nm mean diameter size	Caki-2 cells; IC_50_ value of 0.8 mg/mL	[130]
3. *Rosmarinus officinalis* L. (Leaf extract, H_2_O)	Spherical shape; 100 nm mean diameter size	4T1 cells and C26 cells; IC_50_ values of 44 µg/mL and 100 µg/mL	[131]
4. *Punica granatum* (Fruit peel extract, H_2_O)	Spherical and cubic shape; 26.5 nm mean diameter size	NPC cells and HONE1 cells; IC_50_ values of 197.46 and 85.06 μg/mL	[132]
5. *Lawsonia inermis* (Leaf extract, H_2_O)	Spherical and irregular shape; 45.8 nm mean diameter size	MCF-7 cells; IC_50_ value not available	[133]
6. *Piper betel* (Leaf extract, H_2_O)	Cubic shape; 25.8 nm mean diameter size	A549 cells; IC_50_ value of 104.6 mg/mL	[134]
7. *Garcinia mangostana* (Fruit peel extract, crude)	Spherical shape; 13.4 nm mean diameter size	HCT116 cells and CCD112 cells; IC_50_ values of 99.80 µg/mL and 140.80 µg/mL	[135]

**Table 4:** Anticancer activity of various biosynthesized titanium oxide nanoparticles.

Plant Source	Physicochemical Properties	*In Vitro* Anticancer Activity	References
1. *Terminalia chebula* (Fruit rind extract)	Irregular shape; 80–100 nm diameter size range	A549 cells; IC_50_ value of 62.5μg/mL	[148]
2. *Cinnamomum tamala*, (Leaf extract, H_2_O)	Irregular shape; 23 nm mean diameter size	D145 cells; IC_50_ value not available	[149]
3. *Aloe vera* (Gel extract)	Irregular shape; 11 nm mean diameter size	A549 cells; IC_50_ of values of 165 μg/mL and 210 μg/mL	[150]
4. *Acacia nilotia* (Aerial extract, H_2_O)	Spherical shape; 20–40 nm diameter size range	MCF-7 cells; IC_50_ value not available	[151]
5. *Fomes fomentarius* (Mushroom extract, H_2_O)	Irregular shape; 100–120 nm diameter size range	HCT-116 cells; IC_50_ value not available	[152]
6. *Withania somnifera* (Root extract, H_2_O)	Spherical shape; 50–90 nm diameter size range	HepG2 cells; IC_50_ value of 53.65 µg/mL	[153]
7. *Ledebouria revoluta* (Bulb extract, H_2_O)	Spherical and tetragonal shape; 47 nm mean diameter size	A549 cells; IC_50_ value of 53.65 µg/mL	[154]
